# Recommendations for the re-analysis of existing genomic data from patients with epilepsy

**DOI:** 10.1016/j.gimo.2026.104494

**Published:** 2026-09-10

**Authors:** Alan J. Robertson, Natalie B. Tan, Pei Dai, Carmen Bennett, Clair Sullivan, Zornitza Stark, Lata Vadlamudi, Nicola Waddell

**Affiliations:** 1 Faculty of Medicine, University of Queensland, Brisbane, QLD, Australia; 2 Medical Genomics Group, QIMR Berghofer Medical Research Institute, Brisbane, QLD, Australia; 3 Queensland Digital Health Centre, University of Queensland, Brisbane, QLD, Australia; 4 Sullivan Nicolaides Pathology, Brisbane, Australia; 5 Murdoch Children’s Research Institute, Parkville, VIC, Australia; 6 Department of Paediatrics, Melbourne Medical School, The University of Melbourne, Parkville, VIC, Australia; 7 Victorian Clinical Genetics Services, Murdoch Children's Research Institute, Royal Children's Hospital, Parkville, VIC, Australia; 8 Precision Immunology Program, Garvan Institute of Medical Research, Sydney, NSW, Australia; 9 Department of Immunology, Nepean Hospital, Sydney, NSW, Australia; 10 Virtus Diagnostics, Sydney, NSW, Australia; 11 Frazer Institute, The University of Queensland, Brisbane, QLD, Australia; 12 Department of Neurology, Royal Brisbane & Women's Hospital, Brisbane, QLD, Australia; 13 Metro North Hospital and Health Service, Department of Health, Queensland Government, Brisbane, QLD, Australia; 14 Australian Genomics, Melbourne, VIC, Australia; 15 University of Melbourne, Melbourne, VIC, Australia

**Keywords:** Clinical recommendations, Epilepsy, Genetic testing, Re-analysis intervals, Re-analysis recommendations

## Abstract

Genomic testing is being recognized as an integral part of the management of individuals with epilepsy. However, despite the increasing utility of genomic testing in epilepsy, a substantial proportion of patients who are suspected to have a monogenic cause do not receive a molecular diagnosis. Reanalyzing existing genomic data increases the molecular diagnostic yield over time. As our understanding of the molecular components of epilepsy is changing faster than that of many other conditions, the re-analysis of existing genomic data from patients with epilepsy holds considerable potential for improving diagnostic outcomes.

Given the complexity involved in the re-analysis of existing genomic data, neurologists may be unsure how to best incorporate this into their clinical practice following an initial uninformative genomic result. Here, we describe recommendations specifically designed to guide the re-analysis of existing genomic data for individuals with epilepsy with an unknown etiology. We synthesize current evidence and apply these findings to the specifics of genetic epilepsy to determine the best approaches to integrate re-analysis into clinical practice in a sustainable manner that maximizes benefits to patients.

## Introduction

Epilepsy is a prevalent group of neurological disorders that affect more than 50 million people worldwide.^1^^,^^2^ Known acquired causes of epilepsy include infection, trauma, and stroke.^1^, ^2^, ^3^, ^4^, ^5^ However, genetics is likely to play a role in more than two-thirds of cases where the etiology remains unknown.^6^^,^^7^ For many individuals with epilepsy, their condition is thought to have complex, multifactorial genetic causes; however, a substantial fraction of severe epilepsies are believed to have monogenic causes (up to 40%^8^).

Molecular testing is being increasingly integrated into clinical care.^9^^,^^10^ Molecular testing can provide information that is transformative for affected individuals. A molecular diagnosis can provide new insights into the condition’s natural history and prognosis, lead to more accurate genetic counseling, and help patients and their families make informed reproductive decisions.^11^ For patients with monogenic pathogenic variants, this information has the potential for precision-based therapeutic options as well as further insights into comorbidities and their overall management.^9^

Genomic testing now refers to comprehensive, high-throughput sequencing of a patient's DNA. It is possible to analyze the patient’s sequencing data agnostically or with a specific hypothesis in mind. This allows clinicians to request re-analysis of previously generated genomic data to answer the original clinical inquiry or to investigate separate clinical questions.^12^ For our purposes, genomic testing can be divided into 2 broad categories: exome sequencing and genome sequencing. Exome sequencing focuses on the coding regions of the genome, while genome sequencing aims to sequence every nucleotide in the patient's genome, encompassing both coding and noncoding regions.^12^

Prior to the availability of high-throughput sequencing technologies that made genomic testing possible, a patient's DNA was examined using lower-throughput approaches that probed a patient's DNA in a targeted “gene-by-gene” manner via techniques such as Sanger sequencing.^13^ Here, we refer to this approach as genetic testing. Genetic testing examines a smaller number of predetermined loci and provides a cost-effective method to study a patient's DNA when there is a clear hypothesis in mind (for example, in patients with additional clinical features that indicate underlying causes such as tuberous sclerosis). Genetic testing can include tests that examine a single gene or a small number of loci; however, multigene panels (MGP) make it possible for genetic tests to examine hundreds of genes known to be associated with a specific condition. Both genetic and genomic testing options have a range of advantages and disadvantages (Table 1). However, it is important to recognize that even those with a strong suspicion of a genetic cause for their epilepsy often do not receive a molecular diagnosis after genetic or genomic testing.^14^, ^15^, ^16^

**Table 1:** Tests available to observe the genetic differences in genes associated with patients with epilepsy without a known etiology

Test Type	Summary	Advantages	Disadvantages
Targeted sequencing including multigene panels (genetic testing)	• Sequence the DNA from a defined list of epilepsy genes or loci • Generally focused on coding regions • Assess DNA for any causative variants	• Often the cheapest test • Able to reexamine variants in previously sequenced genes during re-analysis • Small variants and exon-level copy number changes • Testing/analysis/data storage straightforward	• Unable to examine clinically relevant genes and regulatory regions that were not amplified during the initial sequencing experiment • Unable to assess copy number or many large chromosomal structural changes at the same resolution as genomic testing
Exome sequencing (genomic testing)	• Sequence the DNA from protein-coding regions in the entire genome • Determine variants in these regions • Potentially focus on variants from a virtual list of epilepsy genes	• Cheaper than genome sequencing • Able to reexamine variants in previously sequenced genes during re-analysis • Able to examine novel epilepsy genes during re-analysis • Possible to assess chromosomal-level and gene-level copy number changes • Supports secondary uses of data, such as pharmacogenomics	• Generally unable to examine variants outside of protein-coding sequences (eg, regulatory regions, novel exons, and noncoding regions) • Coverage not uniform (notably with exon 1), can affect variant identification • PCR amplification: challenging regions may not produce enough data to call pathogenic variants reliably • Data handling more complex • Large number of identified variants makes variant prioritization more challenging • More challenging to assess structural chromosomal changes • Increased chance of incidental findings
Genome sequencing (genomic testing)	• Sequence the DNA from all protein-coding and noncoding regions across the entire genome • Identify variants across entire genome • Potentially focus on variants from a virtual list of epilepsy genes	• Able to reexamine variants in previously sequenced genes during re-analysis • Able to examine new epilepsy genes during re-analysis • Able to examine variants outside of protein-coding sequence (eg, regulatory regions, novel exons, and noncoding regions) • Able to examine all classes of variants, including short tandem repeats and mitochondrial variants • Supports secondary uses of data, such as pharmacogenomics, and potentially, the future calculation of epilepsy risk burden • Coverage more uniform than exome sequencing	• Most expensive type of testing • Data handling more complex • Large number of identified variants makes variant prioritization complex • Increased chance of incidental findings

*PCR,* polymerase chain reaction.

Re-analysis of existing genomic data is an effective way of providing more patients with a molecular diagnosis.^11^^,^^17^^,^^18^ Since 2016, more than 40 studies have independently shown that reanalyzing existing genomic data improves diagnostic yield over time.^11^^,^^17^^,^^18^ Multiple systematic reviews have shown that reanalyzing genomic data 2 to 3 years after the initial test can increase the diagnostic yield of a test by 10% to 15% by identifying the causative monogenic variant.^17^^,^^18^

The re-analysis of existing information from genetic and genomic tests holds significant clinical potential. While re-analysis of existing genome data has been shown to be more cost-effective than resequencing,^19^ there are concerns about the cost and workload implications required to incorporate routine re-analysis as a standard of care.^11^ Many clinical and laboratory services have adopted a 24- to 36-month re-analysis interval as an informal, pragmatic standard.^17^^,^^18^ This one-size-fits-all approach allows individuals to benefit from re-analysis while working within the finite resources of health care systems.^11^

The underlying genomic architecture of neurological disorders such as epilepsy is complex; however, the application of genomic testing is increasing our understanding of the genetic causes of these conditions.^14^^,^^20^^,^^21^ Previous work from our group revealed that knowledge of the molecular components of epilepsy is changing significantly faster than that of other similar conditions.^20^^,^^22^ Studying the individual genes in a repository of virtual gene panels revealed that the panel used to investigate genetic epilepsy gained 244 new diagnostic genes over a 2.5-year period. When compared with the median number of genes added to similar panels during the same period (27 new diagnostic genes), these results strongly suggest that individuals with epilepsy who are suspected to have a monogenic cause and remain undiagnosed after initial testing may benefit from an approach to re-analysis that has been tailored to the specific attributes of their condition.^20^

Some neurologists are comfortable with this level of change; however, others may feel uncertain about the best approach to reanalyze genomic data from their patients. This uncertainty is compounded by a need for neurologists to have access to specialized training in the effective use of genomic testing and the availability of clinical genetic services. To bridge this gap, we synthesize the current evidence for re-analysis and present a series of recommendations, particularly focusing on individuals with epilepsy without a known etiology who have not received a molecular diagnosis after initial testing but are still suspected of having a monogenic cause for their disease (Table 2). While these recommendations are primarily designed to support neurologists, genetic counselors, and clinical geneticists, we also provide a list of supplementary suggestions for others involved in the diagnostic pathway, including laboratories, health services, and policymakers, as well as data repositories and individual patients (Supplemental Table 1-4).

**Table 2:** Recommendations for the re-analysis of genomic data from individuals with epilepsy without a known etiology

Recommendation	Summary
1	Re-analysis of genomic data should be considered a routine part of genomic testing for patients suspected to have a monogenic cause but without a known etiology who remain without a molecular diagnosis after testing.
2	The potential for re-analysis should be discussed with patients undergoing genomic testing as part of consent and return of results.
3	Where possible, exome or genome sequencing should be preferred over more limited types of genetic testing, because these approaches better facilitate re-analysis.
4	Clinicians should be aware of the limitations of different testing approaches and consider different types of re-analysis depending on context.
5	Updated clinical information from the patient should be communicated to laboratories in the re-analysis request.
6	Current evidence suggests that individuals with a suspected monogenic cause and without a molecular diagnosis may benefit from a 12-month re-analysis interval.
7	Specialized clinical-laboratory genomics MDT meetings can be valuable in clarifying uncertainty about result interpretation and reinterpretation for individuals without a molecular diagnosis.

*MDT,* multidisciplinary team.

### Recommendation 1: Re-analysis of genomic data should be considered a routine part of genomic testing for patients suspected to have a monogenic cause but without a known etiology who remain without a molecular diagnosis after testing

Among the different types of data collected in medicine, genomic data are unique. This is in part due to the size of the genome,^23^ the number of genes in the human genome,^24^ and the volume of genetic variants present in each individual’s genome.^25^ While large, complex datasets are not uncommon in medicine, what sets genomic data apart is our changing understanding of the clinical significance of an individual’s genetic information.^11^^,^^17^^,^^26^ The refinement of existing, and the discovery of new, gene-disease and variant-disease associations have been identified as a key driver of new diagnoses during the re-analysis process in multiple studies (Figure 1).^11^^,^^17^

**Figure 1:**
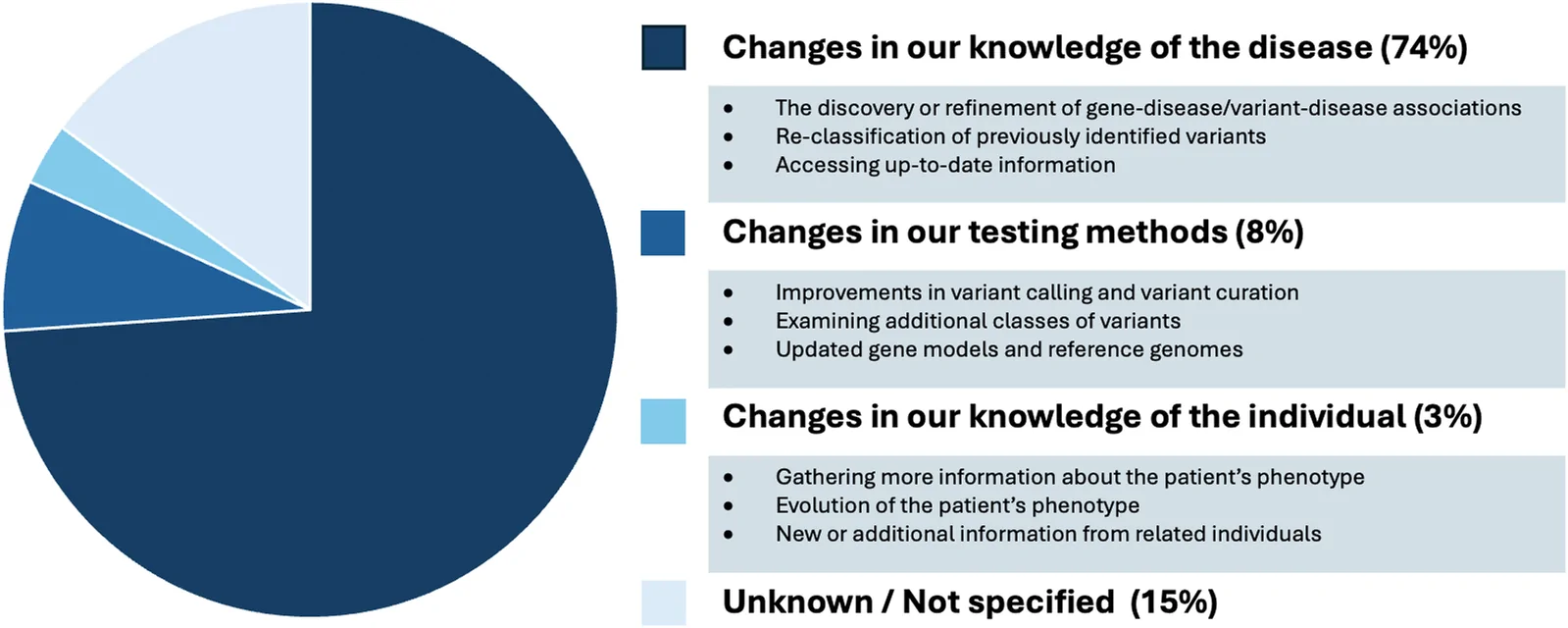
**The mechanisms driving new diagnoses when existing genomic data are reanalyzed and their frequency for delivering new diagnoses.** Each color represents a category of mechanism responsible for increasing diagnostic yield via re-analysis. The data presented here have been adapted from Dai et al^17^ and Robertson et al.^11^

Within the field of epilepsy, the importance of using updated epilepsy gene panels based on the latest evidence was highlighted as early as 2016 by Helbig et al.^15^ However, these are not the only drivers of new diagnoses: changes in our understanding of an individual’s clinical phenotype and changes to laboratory methods are both important in delivering new diagnoses through re-analysis.^11^^,^^17^ To maximize the likelihood of a patient receiving a molecular diagnosis, the re-analysis of existing genomic data should be considered part of genomic testing and, therefore, routine clinical and diagnostic practice.

### Recommendation 2: The potential for re-analysis should be discussed with patients undergoing genomic testing as part of the consent process and return of results

The importance of change in uncovering new diagnoses at a later time can be a challenging concept for patients, because it does not apply to many other types of diagnostic tests.^27^^,^^28^ As a result, we recommend that neurologists spend additional time to ensure that the discussion of genomic testing with patients covers how changes to our understanding of genomic data can affect the results of their test and the capacity for reanalyzing their data in the future to identify clinically important changes. This process ensures that individuals with epilepsy are able to provide informed consent to re-analysis, whether that be at the time of original testing and/or upon revisiting this specific re-analysis need at a subsequent time point, while recognizing that consent practices differ globally.

To this end, consent forms and accompanying information about genomic testing should explicitly state that the interpretation of genomic data can change over time and how that may affect the patient. Recent work by Outram et al^28^ highlights the need to ensure that patients understand the triggers to initiate re-analysis and the potential timings for re-analysis. While this work was centered on groups from underserved populations, these findings are likely universal.

### Recommendation 3: Where possible, exome or genome sequencing should be preferred, because these approaches better facilitate re-analysis

In the last 2 decades, advances in genome sequencing technologies have made genomic testing a clinical reality and have placed the entirety of the patient's genetic information within the clinician's reach.^1^^,^^2^^,^^9^^,^^28^^,^^29^ Genomic tests, much like genetic tests, have advantages and disadvantages (Table 1), and consequently, the choice of tests can affect how the patient’s data can be reanalyzed.

A key advantage of genomic testing is the potential to use virtual gene panels. While not an essential component of genomic testing, virtual gene panels are evolving lists of genes known to be clinically associated with a condition and provide a means to manage our changing understanding of the genetic components of a disease^30^, ^31^, ^32^ while staying focused on the clinical indication for testing. As new clinically significant information is discovered, and our understanding of the specific disease mechanisms matures, genes can be added to (and removed from) a virtual gene panel. This approach enables laboratories to focus on the genes known to be associated with a condition at the time of testing. Updated versions of a virtual gene panel can be used during re-analysis to examine genes that were subsequently discovered after the initial testing.^31^^,^^32^ As a result, genomic testing with virtual gene panels allows clinicians and laboratories to use the most up-to-date, evidence-based information to analyze a patient’s DNA, irrespective of when the patient’s DNA was sequenced. However, it is also important to recognize that genome sequencing can be significantly more expensive and resource-intensive when compared with more traditional forms of genetic testing.^11^^,^^33^ As a result, genomic testing is not available to all patients. To this end, it is important to consider the re-analysis of the data produced by genetic tests, such as MGPs.

Both genetic and genomic tests can uncover candidate variants that do not quite meet evidence-based criteria to be classified as disease-causing.^11^^,^^17^^,^^34^ In some cases, additional information emerges over time that provides the evidence required to definitively classify a variant as disease-causing. Reassessing these variants, whether they were identified by genome sequencing or genetic testing, can still produce additional diagnoses. The key benefit of genomic testing over the most sophisticated MGPs is that genomic tests can examine additional loci not known to be associated with the condition at the time of test design, while still providing an opportunity to reassess the variants identified during the original analysis.

As a result, we recommend that neurologists prefer genomic testing over genetic testing and, when possible, consider genome sequencing over exome sequencing. Genomic approaches generate more comprehensive data that can be reanalyzed in additional ways (Table 1). While genomic testing might not currently be available to all health services, the advent of new technologies promising sub-$100 human genomes^35^^,^^36^ will allow more health systems to eventually move toward genome-based testing practices.

### Recommendation 4: Clinicians should be aware of the limitations of different testing approaches and consider different types of re-analysis depending on context

Genome analysis spans a range of different processes, each requiring various methods to produce and analyze different types of data. Consequently, the re-analysis of these data can include a range of different approaches and methodologies;^11^^,^^37^ depending on a patient’s genomic data and the types of advancements available, a patient's existing genomic data can be reanalyzed in various ways.^29^ For example, the curation processes involved in reinterpreting a candidate variant of uncertain significance in light of new phenotype information or new literature reports would differ from the bioinformatic processes needed to interrogate a number of newly published disease genes.^11^

The processes involved in re-analysis can take many forms, and while it is important for neurologists to recognize this, they should not be expected to memorize particulars such as the subtle differences between reference genomes, variant callers, and the genes included in specific versions of specific panels available at specific points in time. Instead, we recommend that neurologists recognize that there are limits to even the best genomic tests and comprehensive epilepsy gene lists,^38^ and, when the situation warrants it, develop the capacity to engage with laboratories and/or clinical genetics subspecialists to inquire about the additional, complementary, or expanded tests that could be ordered to further examine a patient’s genome. Given their experience with these tests, laboratories are ideally positioned to provide guidance (Supplemental Box 1); however, a clinical genetics subspecialist may provide a valuable, alternative opinion.

### Recommendation 5: Updated clinical information from the patient should be communicated to laboratories in the re-analysis request

Neurologists are attuned to recognizing changes in their patients’ clinical phenotype and exploring relevant family history information. However, laboratories are not always made aware of this updated information. Because these findings can be key to making sense of genomic variants, it is important that neurologists have clearly defined pathways to communicate these updates to the testing laboratories, so that these groups can best reanalyze an individual’s genomic data within the context provided by newly discovered genes in light of the additional phenotypic data.

Conversely, neurologists should also ensure that patients understand the importance of staying in contact with their health care providers. Care should be taken to highlight that clinically significant changes in themselves or in family members can be critically important for understanding their condition. For some families, this evolving phenotypic information can provide the “missing link” for laboratories to properly contextualize their genetic information and make a formal molecular diagnosis (Supplemental Box 4).

### Recommendation 6: Current evidence suggests that individuals with a suspected monogenic cause and without a molecular diagnosis may benefit from a 12-month re-analysis interval

Waiting 24 to 36 months after the initial analysis has emerged as an informal standard for reanalyzing existing genomic data.^17^^,^^18^ This approach has several clear benefits; it is pragmatic, gives clear instructions, and provides a simple “rule of thumb”. While this interval increases the workload placed on laboratories, the impact is more manageable than that with more frequent re-analysis intervals.^39^ The 24- to 36-month interval may be ideal for some conditions; however, previous studies that have examined the clinical potential of re-analysis for epilepsy have used shorter re-analysis intervals. The Epilepsy Genetics Initiative reanalyzed individuals every 6 months,^40^ while Li et al^41^ suggested that a suitable time point for re-analysis of those with epilepsy and intellectual disability fell between 6 and 12 months.

To examine how our understanding of the molecular components of epilepsy is changing over time, we previously described a method to determine the ways in which virtual gene panels have changed over a period of 2.5 years.^20^ Application of this method to 112 virtual gene panels used by Australian laboratories revealed that the Genetic Epilepsy panel gained 244 diagnostic-grade genes—a number significantly higher than the majority of panels designed to examine other conditions (median of 27 genes).

Applying this method to the virtual gene panel used by the National Health Service England Genomic Medicine Service to test patients with epilepsy showed a similarly large increase in the number of diagnostic genes.^22^ However, analysis of the number of genes added to each panel each month revealed stark differences in the way in which genes were added (Figure 2). Unlike the virtual gene panels from PanelApp Australia, which saw new diagnostic genes added throughout the year, the epilepsy panel used by the National Health Service saw annual diagnostic gene updates, resulting in large “spikes” in the number of genes made available to laboratories for analysis (Figure 2).

**Figure 2:**
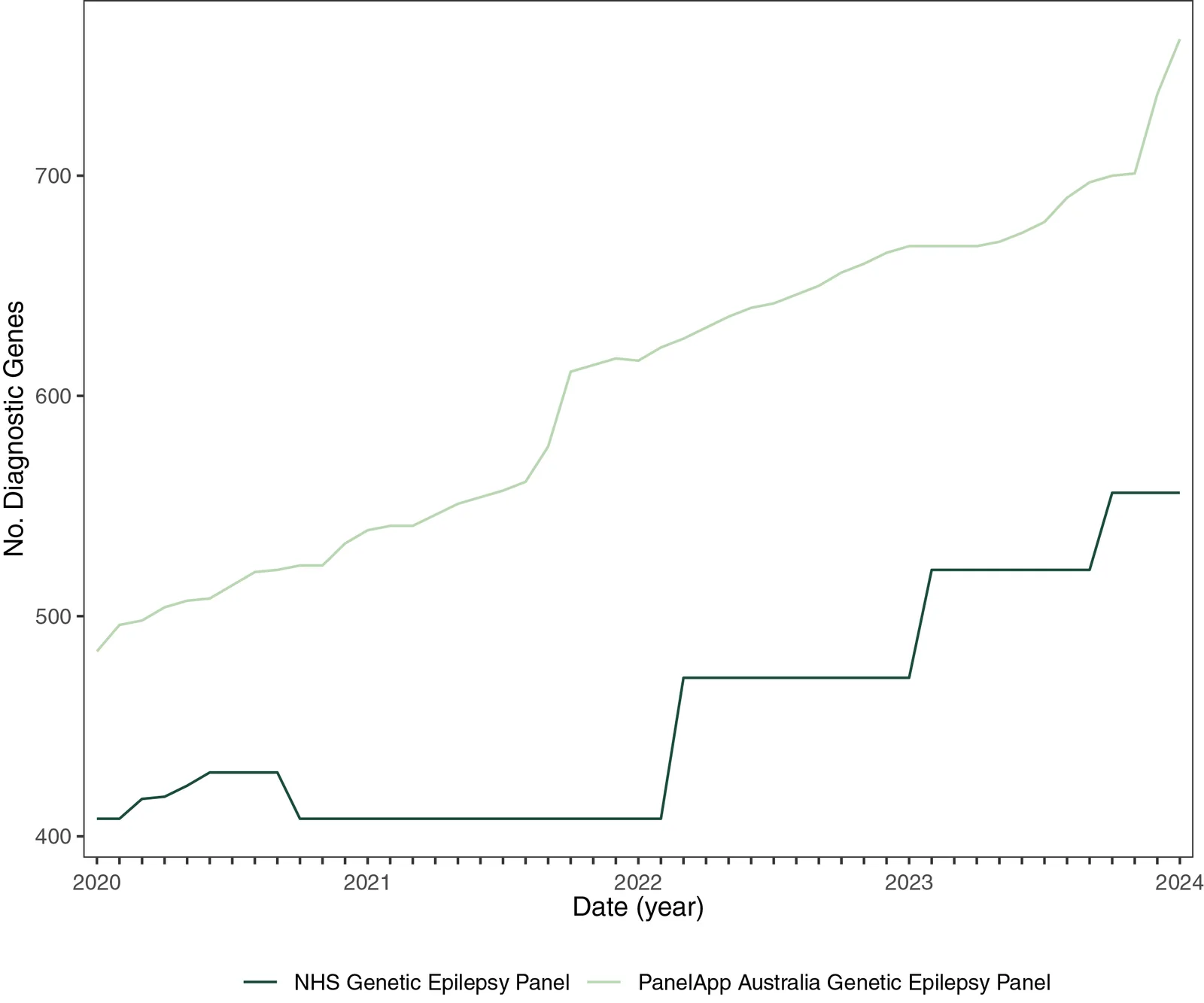
**The number of diagnostic genes each month in epilepsy virtual gene panels between January 2020 and January 2024.** The number of diagnostic, clinical grade genes in the Epilepsy Gene Panel used by the NHS is shown in dark green. The number of diagnostic, clinical grade genes in the Epilepsy Gene Panel used by Australian Laboratories is shown in light green. *NHS,* National Health Service.

Because the discovery of new genes is a key mechanism for the diagnoses made by re-analysis, these results indicate that individuals with epilepsy may benefit from more frequent re-analysis intervals. We, therefore, recommend that patients with epilepsy without a molecular diagnosis and suspected to have a monogenic cause, who have undergone genomic testing, have their genomic data reanalyzed once every 12 months. This recommendation recognizes both the rapid pace of discovery surrounding our knowledge of the molecular components of epilepsy and the labor-intensive and resource-intensive nature of re-analysis. However, given the importance of the discovery of new gene-disease relationships, it is important to highlight that patients who have undergone genetic testing (even with the most comprehensive MGP) may not see the same benefit from this 12-month re-analysis interval as those who have undergone genomic testing.

More frequent or dynamic re-analyses could be triggered by changes in patient phenotype or the addition of a large number of diagnostic genes to virtual gene panels. However, any re-analysis approach needs to consider whether there are means to balance the increase in workload for laboratories, even though re-analysis of existing genomic data is more cost-effective than resequencing. Re-analysis is currently labor- and resource-intensive, and the implementation of any form of frequent re-analysis-enabled practice will require considerable automation.^33^ Such automated approaches are increasingly becoming feasible,^42^ and we anticipate that re-analysis may move toward a more continuous, automated model in the near future.

It is important to highlight that this interval may be revised over time. Outside the adoption of automated re-analysis approaches, the discovery of new clinically significant epilepsy genes may plateau, or future studies may have the information needed to develop a receiver operating characteristic curve and provide more accurate guidance surrounding timing intervals. While these examples demonstrate the evolving nature of this space and the importance of revisiting this recommendation, given the significance of a molecular diagnosis to patients, we believe that this 12-month interval can benefit individuals now.

### Recommendation 7: Specialized clinical-laboratory genomics multidisciplinary team (MDT) meetings can be valuable in clarifying uncertainty about result interpretation and reinterpretation for individuals without a molecular diagnosis

Our understanding of the genetics of disease is changing so rapidly that neurologists may be unable to keep up with the breadth of research, or the new techniques and technologies being developed. To this end, building links to communities with expertise in this space and establishing an ongoing dialogue are essential. In recent years, there has been a proliferation of genomic MDT meetings.^9^^,^^43^ These meetings bring together clinicians (including clinical genetics subspecialists), laboratory scientists, and researchers and allow them to present complex or challenging cases for discussion. Many have found this approach to be beneficial for patients with a range of different conditions.^7^^,^^34^^,^^35^ In situations where there is complexity or uncertainty surrounding a patient’s genotype or phenotype, we recommend presenting this case to a clinical-laboratory genomic MDT that is ideally attended by clinical geneticists and genetic pathologists. We acknowledge that not all neurologists will have access to these meetings, and in these situations, we hope to highlight the potential of case conferences and other multi/trans-disciplinary groups.

## Concluding Remarks

Genomic testing is providing a molecular diagnosis for a portion of patients with epilepsy who previously did not have a cause for their epilepsy.^29^ While this is a significant achievement, we must recognize that a large proportion of individuals with epilepsy, even with a suspected monogenic cause, do not receive a molecular diagnosis after comprehensive genome testing (with estimates suggesting that approximately 58% remain undiagnosed).^10^ However, because a very large number of new epilepsy genes are being discovered each year, individuals with epilepsy are uniquely positioned to benefit from reanalyzing existing genomic data. To help individuals with epilepsy benefit from re-analysis, we provide a series of practical recommendations to assist neurologists in navigating the complexities of this area. Each recommendation supports a different area of practice, from consent and test selection to an epilepsy-specific re-analysis interval and ensuring that neurologists have support in interpreting complex results. While these recommendations were developed to support the analysis of genomic data from patients with epilepsy, the underlying principles are likely to be applicable to other inherited conditions where the pace of gene discovery and the severity of the condition warrant periodic reexamination of existing genomic data.

We have recommended that “individuals without a molecular diagnosis and a suspected monogenic cause, who underwent genomic testing, may benefit from a 12-month re-analysis interval despite the increased workload.” This interval is supported by the existing literature^39^, ^40^, ^41^ and is informed by the specific patterns of change observed in epilepsy gene panels over time.^20^, ^21^, ^22^ A limitation of this approach is that we have not been able to determine the clinical impact of this recommendation. Future iterations of these recommendations would benefit from analysis of how a 12-month re-analysis interval affects the number of patients receiving a molecular diagnosis and the differences between genetic and genomic testing. However, it is important to recognize that while a molecular diagnosis *can* lead to changes in clinical management, such as eligibility for targeted precision therapies, more nuanced counseling, and better-informed reproductive planning, the direct impact of a 12-month re-analysis interval has not been assessed and represents an important area for future studies.

It is important to recognize that while reanalyzing existing genomic data can provide new molecular diagnoses, this process is often complex, costly, and labor-intensive. As a result, for some health systems, a 12-month re-analysis interval may need to be revised. In these scenarios, any re-analysis of patient data is preferable to none. While more expensive than re-analysis of existing data,^19^ some individuals may also benefit from resequencing. The benefits of resequencing should be considered in a case-by-case assessment of the advantages (or not) of performing updated sequencing using the latest assays, because improvements in sequencing chemistries (eg, polymerase chain reaction-based vs polymerase chain reaction-free library preparation) and platforms (eg, short- vs long-read sequencing) can determine the diagnostic gains these new approaches could offer those who were sequenced at an earlier time when technological errors may have been more prevalent.

We also propose a dynamic approach to re-analysis based on the accumulation of new knowledge rather than only on the passage of time. It is important to clarify that re-analysis is not limited to the addition of a large number of clinically relevant genes to a clinical-grade gene panel. For example, dynamic re-analysis could be triggered by the release of a new reference genome or by the release of a new version of a variant caller. While dynamic re-analysis has great potential, the specific triggers for re-analysis must be determined.

Automating re-analysis has been proposed as a solution to the cost and workload challenges associated with the routine manual reexamination of existing genomic data.^42^ Automation would enable large stored datasets to be frequently reanalyzed with minimal impact on the laboratory. However, the development of automated re-analysis pipelines requires a significant investment in establishing, testing, and maintaining a range of processes. While the investment in these systems may be significant, the capacity to provide routine or dynamic approaches to re-analysis can potentially be transformative for patients.

We have attempted to ensure that the recommendations presented here are widely applicable. At this stage, we intend for these recommendations to support both pediatric and adult patients with epilepsy. While monogenic etiologies are more commonly identified in patients with severe, early-onset the evidence collected by PanelApp and PanelApp Australia does not differentiate between these cohorts, and as such, we cannot currently recommend different intervals for these different groups. However, future work may demonstrate the benefits of a stratified approach.

We recognize that genomic testing is not accessible to all patients and that there is variation in the types of re-analysis available to clinicians. However, we are confident that barriers to genomic testing and re-analysis will continue to decrease and that information from patients' DNA will eventually be accessible to neurologists very broadly.

We highlight our changing understanding of the molecular components of epilepsy. While we have attempted to provide a synthesis of the literature and available data to provide a series of actionable recommendations for neurologists wishing to implement re-analysis in their clinical practice, it is likely that new findings may challenge some of these recommendations. To this end, future studies should reassess the recommendations made here and link them to changes in patient outcomes.

## Data Availability

No datasets were generated or analyzed during this study.

## Ethics Declaration

The authors confirm that they have read the Journal’s position on issues involved in ethical publication and affirm that this report is consistent with those guidelines.

## Conflict of Interest

Alan J. Robertson is the founder and director of ClearSKY Genomics. ClearSKY Genomics is developing a genome browser for clinical use. This platform enables clinicians to better visualize genetic variants in their patients’ DNA, and communicate these findings to their patients. The work presented in this manuscript is independent from the work carried out by ClearSKY Genomics. ClearSKY Genomics has no financial interest in the recommendations described here. Nicola Waddell is a co-founder of genomiQa, a spin-out company from QIMR Berghofer. genomiQa specializes in clinical whole genome analysis for patient diagnosis and treatment selection. genomiQa has no financial interest in the recommendations described here.
