# Supplementary material for: Recommendations for the re-analysis of existing genomic data from patients with epilepsy
Source: Genet Med Open. 2026 Sep 10;4:104494. doi: 10.1016/j.gimo.2026.104494 (PMC13734808; doi:10.1016/j.gimo.2026.104494)
Supplement: Supplementary file 1 [file mmc1.docx]

**Supplementary**

**Supplementary Table 1 – Recommendations for laboratories**

| **Recommendation** | **Summary** |
| --- | --- |
| **1** | Internal policies to account for the evolving nature of virtual gene panels should be clearly defined |
| **2** | There should be a specific policy for the initiation of re-analysis |
| **3** | A process to request re-analysis should be available to clinicians |
| **4** | Dedicated pricing for different types of re-analysis should be provided |
| **5** | Policies for re-contacting a clinician when the classification of a previously reported variant changes should exist |
| **6** | Potential complementary molecular testing should be recommended to clinicians where applicable |
| **7** | Laboratories should routinely share results with publicly accessible genome data repositories |
