# Supplementary material for: Recommendations for the re-analysis of existing genomic data from patients with epilepsy
Source: Genet Med Open. 2026 Sep 10;4:104494. doi: 10.1016/j.gimo.2026.104494 (PMC13734808; doi:10.1016/j.gimo.2026.104494)
Supplement: Supplementary file 2 [file mmc2.docx]

**Supplementary Table 2 – Recommendations for health providers and policy makers**

| **Recommendation** | **Summary** |
| --- | --- |
| **1** | The clinical significance of re-analysing existing genomic data must be recognised by policy |
| **2** | There must be funding avenues to support re-analysis |
| **3** | A cost-benefit analysis comparing genetic tests with virtual gene panels should be performed for each health service |
| **4** | The impact of re-analysing a specific condition should be quantified, as differences in practice may impact the cost-benefit relationship |
| **5** | Ensure that there are policies and funding to enable laboratories to share their results with public data repositories |
| **6** | Ensure that clinicians receive the appropriate training (formal or self-directed) on patient education for re-analysis, specifically covering the concept of genetic change, timing intervals, and patient responsibilities |
