# Supplementary material for: Recommendations for the re-analysis of existing genomic data from patients with epilepsy
Source: Genet Med Open. 2026 Sep 10;4:104494. doi: 10.1016/j.gimo.2026.104494 (PMC13734808; doi:10.1016/j.gimo.2026.104494)
Supplement: Supplementary file 3 [file mmc3.docx]

**Supplementary Table 3 – Recommendations for data repositories**

| **Recommendation** | **Summary** |
| --- | --- |
| **1** | Develop functionality to alert users of significant events. For example, alert the users of a panel if the number of diagnostic genes increases by more than 10% since they last logged in |
| **2** | Develop capacity to visualise changes within a database to enable users to identify periods of rapid change |
| **3** | For repositories like PanelApp and ClinGen, encourage the expert users to suggest an evidence-based re-analysis interval for the specific panel |
