# Supplementary material for: Recommendations for the re-analysis of existing genomic data from patients with epilepsy
Source: Genet Med Open. 2026 Sep 10;4:104494. doi: 10.1016/j.gimo.2026.104494 (PMC13734808; doi:10.1016/j.gimo.2026.104494)
Supplement: Supplementary file 4 [file mmc4.docx]

**Supplementary Table 4 – Recommendations for patients awaiting a molecular diagnosis**

| **Recommendation** | **Summary** |
| --- | --- |
| **1** | Our understanding of your results may change |
| **2** | Keep in contact with your doctor; let them know if you relocate |
| **3** | If your condition changes, or if a related individual develops a similar condition, let your doctor know |
| **4** | Ask your doctor how often your results will be re-analysed, and how you will be updated |
